# (*E*)-7-[(4-Nitro­phen­yl)diazen­yl]-3a-(*p*-tol­yl)-2,3,3a,4-tetra­hydro-1*H*-benzo[*d*]pyrrolo­[1,2-*a*]imidazol-1-one 0.58-dimethyl sulfoxide 0.42-aceto­nitrile solvate: crystal structure, Hirshfeld analysis and DFT estimation of the energy of inter­molecular inter­actions

**DOI:** 10.1107/S2056989017013937

**Published:** 2017-09-29

**Authors:** Vyacheslav S. Grinev, Natalya V. Babkina, Alevtina Yu. Yegorova

**Affiliations:** a Institute of Chemistry, N.G. Chernyshevsky National Research Saratov State University, Astrakhanskaya ul. 83, Saratov 410012, Russian Federation; b Institute of Biochemistry and Physiology of Plants and Microorganisms, Russian Academy of Sciences, Prospekt Entuziastov 13, Saratov 410049, Russian Federation

**Keywords:** crystal structure, X-ray diffraction analysis, π–π inter­actions, DFT calculations, energy of π–π inter­actions, Hirshfeld analysis, azo dye

## Abstract

In the crystal structure of the title compound, C_23_H_19_N_5_O_3_·0.58C_2_H_6_OS·0.42C_2_H_3_N, prepared by the azo coupling of the 4-nitro­phenyl­diazo­nium salt with 3a-(*p*-tol­yl)-2,3,3a,4-tetra­hydro-1*H*-benzo[*d*]pyrrolo­[1,2-*a*]imidazol-1-one, the azo mol­ecules are linked by N—H⋯O hydrogen bonds into chains along the *a*-axis direction, and by the π–π inter­action into [101] chains. The dimethyl sulfoxide and aceto­nitrile solvent mol­ecules occupy the same positions, with populations of 0.585 (3) and 0.415 (3), respectively. These mol­ecules take part in C—H⋯O(N) and C—H⋯π contacts. The energy of the π–π inter­actions was estimated using DFT calculations. The Hirshfeld mol­ecular surface analysis revealed the positions of the most important inter­molecular contacts, such as hydrogen bonds and π–π inter­actions.

## Abstract 2

In the crystal structure of the title compound, C_23_H_19_N_5_O_3_·0.58C_2_H_6_OS·0.42C_2_H_3_N, prepared by the azo coupling of the 4-nitro­phenyl­diazo­nium salt with 3a-(*p*-tol­yl)-2,3,3a,4-tetra­hydro-1*H*-benzo[*d*]pyrrolo­[1,2-*a*]imidazol-1-one, the azo mol­ecules are linked by N—H⋯O hydrogen bonds into chains along the *a*-axis direction, and by the π–π inter­action into [101] chains.

## Chemical context   

Compounds prepared by azo coupling of aryl­diazo­nium salts with 3*a*-aryl-2,3,3*a*,4-tetra­hydro-1*H*-benzo[*d*]pyrrolo­[1,2-*a*]imidazol-1-one (**1**) are crystalline substances with deep color varying from yellow to red, depending on the structure of the initial diazo­nium cation. Since several nucleophilic centers in **1** can be attacked by the electrophilic diazo­nium cation, it was of inter­est to study the effect of heteroatoms, as well as other mol­ecular fragments, on the mol­ecular reactivity. The presence of the secondary amino group allows the formation of triazene derivatives. However, the most likely site of electrophilic attack is a fused aromatic ring activated by N heteroatoms. The azo dye mol­ecules constructed in this way can exist in two forms, *E* and *Z*, depending on the presence or absence of certain stabilizing factors: bulky substituents, intra­molecular hydrogen bonds, non-covalent inter­actions, *etc*. One of the representatives of the synthesized series is 7-[(4-nitro­phen­yl)diazen­yl]-3a-(*p-*tol­yl)-2,3,3a,4-tetra­hydro-1*H*-benzo[*d*]pyrrolo­[1,2-*a*]imidazol-1-one (**2**), which was prepared from 4-nitro­phenyl­diazo­nium chloride and **1**. For the final determination of the structure of the azo product, an X-ray diffraction study of a crystal grown from DMSO–aceto­nitrile solution as a mixed DMSO/aceto­nitrile solvate of **2** was performed.
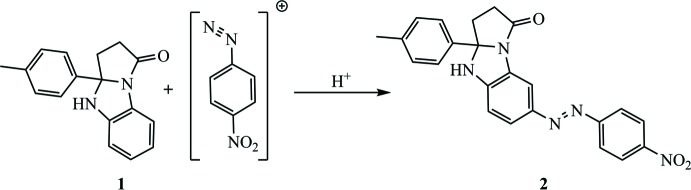


## Structural commentary   

**Figure 1:**
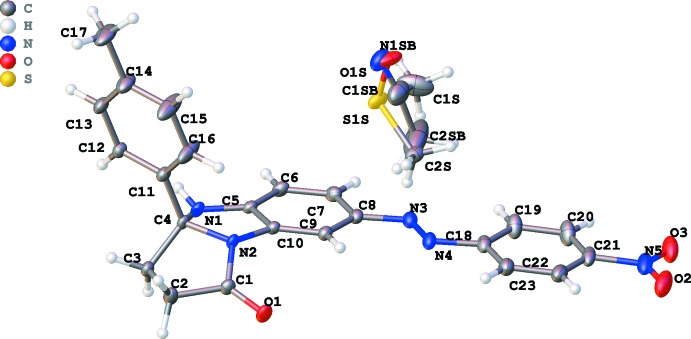
The asymmetric unit of the title compound with overlapping solvent mol­ecules of DMSO and aceto­nitrile. Displacement ellipsoids are drawn at the 50% probability level.

The asymmetric unit of the title compound is shown in Fig. 1 ▸. The mol­ecules of **2** have the *E*-configuration that was expected because of the *para* position of the nitro group in the aryl­diazenyl fragment. Part of the mol­ecule of **2**, including the 4-nitro­phenyl and benzimidazole fragments linked by the azo group, is close to planar, with the dihedral angle formed by two aromatic rings being 2.73 (7)°. The largest deviation from the mean plane of the benzimidazole ring system is 0.1300 (9) Å for C4. The 1*H*-imidazole ring adopts an envelope conformation with C4 atom as the flap, thus introducing some non-planarity into the conjugated part of the mol­ecule. The pyrrolidone ring is twisted with respect to the C2—C3 bond, thus the environment of the N2 amide atom becomes non-planar and this atom deviates by 0.267 (1) Å from the plane formed by the three neighboring C atoms. As as result, the C1—N2 distance [1.3737 (17) Å] is larger than average for γ-lactams [1.347 (14) Å; Allen *et al.*, 1987 ▸]. The relatively long N2—C10 distance [1.4091 (17) Å] indicates weak π-conjugation and gives an insight into why substitution takes place at the 8 position.

## Supra­molecular features   

**Figure 2:**
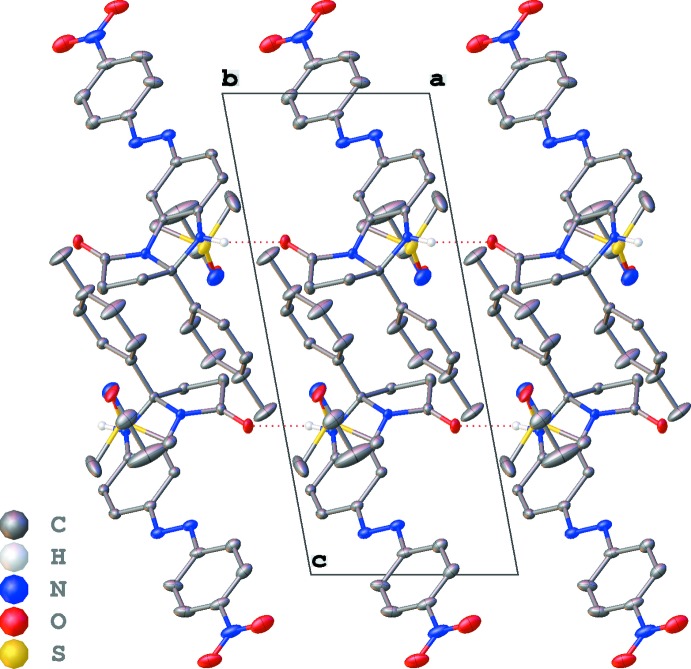
The packing diagram viewed along the *b* axis. N—H⋯O hydrogen bonds are represented by dotted lines.

**Figure 3:**
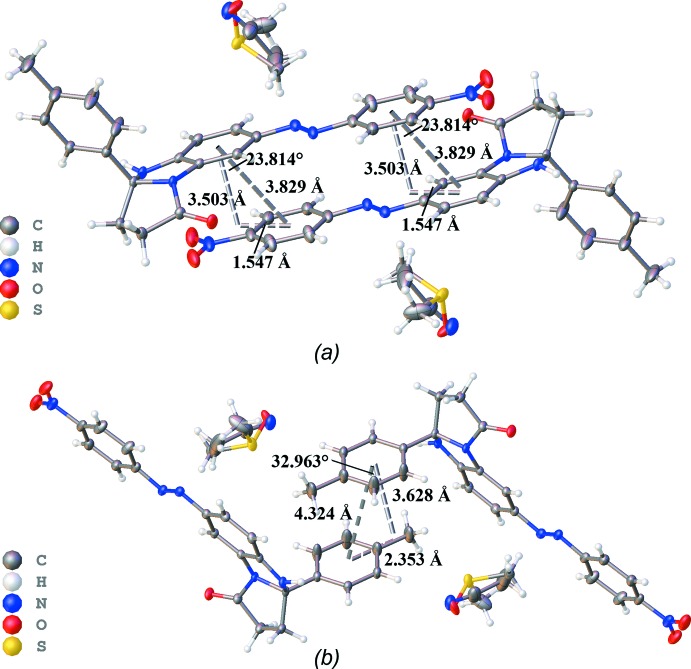
Diagram showing π–π inter­actions between mol­ecules of **2** (*a*) between the aromatic rings of the benzimidazole group and the 4-nitro­phenyl substituent, (*b*) between the aromatic rings of two *p-*tolyl substituents.

In the crystal, mol­ecules of **2** are linked by N—H⋯O hydrogen bonds into chains along the *a-*axis direction (Table 1 ▸, Fig. 2 ▸). These mol­ecules are also linked by π–π inter­actions between the aromatic rings of the benzimidazole fragments and 4-nitro­phenyl substituents as well as between *p*-tolyl substituents (Table 2 ▸, Fig. 3 ▸), thus forming chains along the [101] direction. Comparing geometric parameters related to these π–π inter­actions (Table 2 ▸), one can conclude that those involving *p*-tolyl substituents are weaker. The di­methyl­sulfoxide and aceto­nitrile solvent mol­ecules occupy the same positions with populations of 0.585 (3) and 0.415 (3), respectively. These mol­ecules participate in inter­molecular inter­actions as donors of H-atoms of the methyl groups of aceto­nitrile and DMSO, and as H-atom acceptors *via* the electronegative O and N atoms (Table 1 ▸).

## Hirshfeld surface analysis   

**Figure 4:**
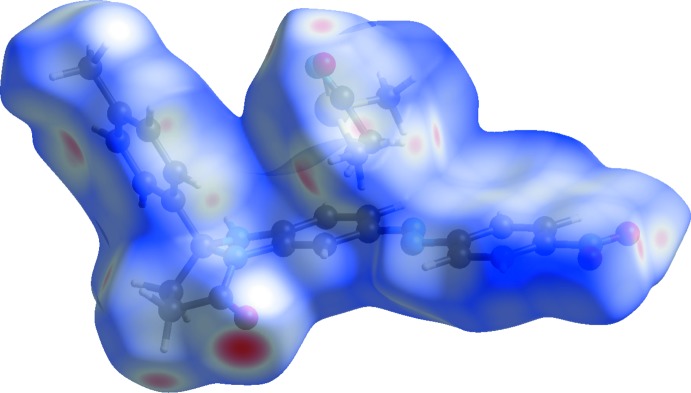
Hirshfeld surface diagram for the asymmetric unit of the title compound.

**Figure 5:**
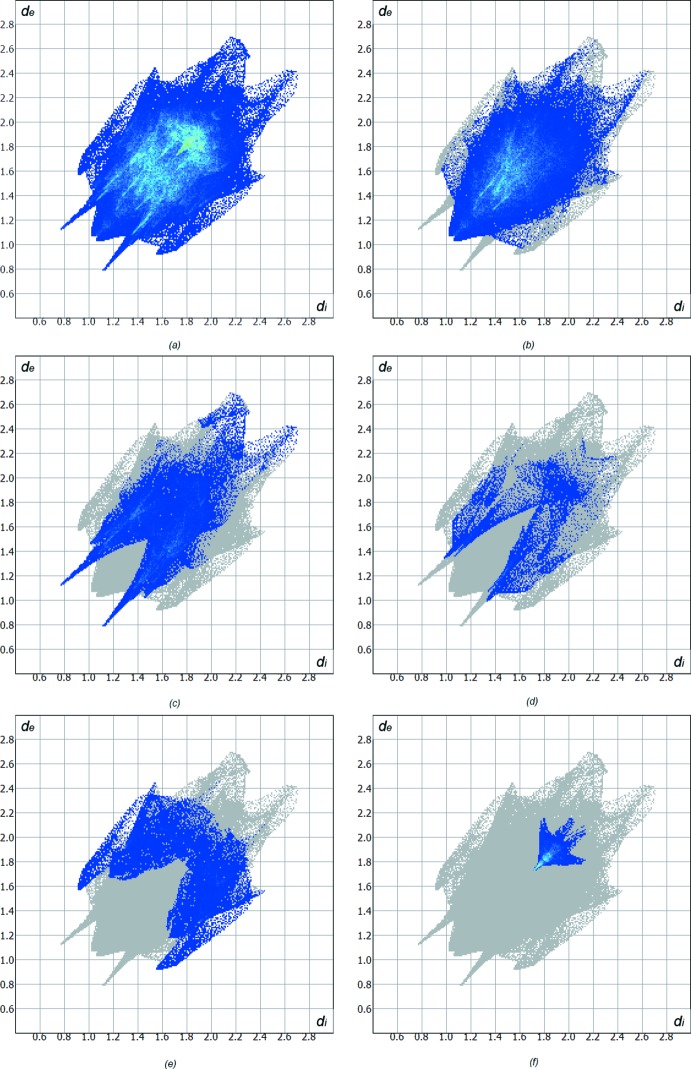
Diagrams showing (*a*) the full two-dimensional fingerprint plot, and those delineated into (*b*) H⋯H, (*c*) O⋯H/H⋯O, (*d*) N⋯H/H⋯N, (*e*) C⋯H/H⋯C, and (*f*) C⋯C contacts.

Hirshfeld surface analysis (Hirshfeld, 1977 ▸) of the title crystal structure allows us to visualize inter­molecular inter­actions. The contribution of the H⋯H inter­molecular inter­actions amounts to 47.6%. The contributions of other important inter­actions are as follows: H⋯O (21.2%), H⋯C (11.2%) and H⋯N (5.1%). Other contacts C⋯O (3.9%), C⋯C (3.8%), C⋯N (3.6%), and H⋯S (2.1%) are less than 5%. The Hirshfeld surface diagram, *d*_norm_, with transparency (Fig. 4 ▸), indicates (in red) locations of the strongest inter­molecular contacts with participation of atoms H6*A*, H2*A* and H2*B* (Fig. 4 ▸). The H⋯H, H⋯C, H⋯S and H⋯O contributions to the crystal packing are shown as two-dimensional fingerprint plots with blue dots (Fig. 5 ▸). The *d_e_* (*y* axis) and *d_i_* (*x* axis) values represent the closest external and inter­nal distances (Å), respectively, from the given points on the Hirshfeld surface (Wolff *et al.*, 2012 ▸). The inter­molecular hydrogen bonds are indicated by the H⋯O contacts (21.2%) on the dotted diagram (Fig. 5 ▸ *c*). Two sharp spikes with *d_e_* + *d_i_* = ∼2.0 Å visualize the experimentally obtained value of 2.04 (2) Å for the H⋯O distance corresponding to a hydrogen bond between azo mol­ecules. The C⋯C contacts (3.8%) reflect π–π inter­actions between the mentioned above aromatic rings (Figs. 4 ▸, 5 ▸ *f*). In addition, there are some H⋯π contacts (H⋯C), which are mostly located at hydrogen atoms of the CH_3_ group of the *p*-tolyl substituent of one mol­ecule and the π-system of the same substituent of the neighboring mol­ecule (Fig. 5 ▸ *e*).

## Quantum chemical DFT calculations   

To compare the energies of the two types of inter­molecular π–π inter­actions found in the title crystal, we performed quantum chemical modeling of this system at the level of Density Functional Theory (DFT). All DFT calculations were made using GAUSSIAN09 package (Frisch *et al.*, 2010 ▸) and high-performance computing cluster of National Research Saratov State University. Crystallographic coordinates were used as a starting point, and full geometry optimization of monomer and dimers was performed using an mPW1B95 functional with a 6-31g(d) basis set. This hybrid meta density functional theory (HMDFT) method based on the modified Perdew and Wang exchange functional (mPW) and Becke’s 1995 correlation functional (B95) gives good results for the systems with non-covalent inter­actions, such as hydrogen bonding and weak van der Waals inter­actions (Zhao & Truhlar, 2004 ▸). The energy of the π–π inter­action was estimated using the following simple equation:

*E_inter­action_* = *E_dimer_* – 2 × *E_monomer_*

A comparison of some parameters of non-covalent inter­actions for the optimized geometry of **2** and for the crystallographic data is presented in Table 2 ▸. The chosen level of theory reproduces the geometrical parameters of the inter­molecular inter­actions quite well. Thus, the energies of π–π inter­actions of both types, between the aromatic rings of the benzimidazole fragment and of the 4-nitro­phenyl substituent and between the two aromatic *p-*tolyl substituents at the 3*a* positions, can be estimated to be equal to −16.5 and −3.0 kcal mol-1, respectively.

## Database survey   

Mol­ecule **2** may be considered as being composed of two fragments, a heterocyclic core and the 4-nitro­phenyl­diazenyl substituent. The latter is relatively abundant and a search in the Cambridge Structural Database (CSD, Version 5.37, update May 2016; Groom *et al.*, 2016 ▸) returned eight hits [CSD refcodes: EMAWUL (Yazıcı *et al.*, 2011 ▸), KEMFUE (Centore *et al.*, 2006 ▸), LEZXAQ and LEZXUK (Šimůnek *et al.*, 2007 ▸), PIDVAA (Kasyan *et al.*, 2007 ▸), ROMNIR (Lu *et al.*, 2009 ▸), TIVBOQ (Rodriguez *et al.*, 2008 ▸), YEDYIQ (You *et al.*, 2006 ▸)], but no heterocyclic compounds were found among them. The closest to the heterocyclic core of **2** is the previously reported 3*a*-phenyl-2,3,3*a*,4-tetra­hydro-1*H*-pyrrolo­[1,2-*a*]benz­imidazol-1-one (CSD refcode CIGPEN01; Grinev and Egorova, 2013 ▸). Other examples of compounds containing the same heterocyclic core are disubstituted at the 2 position: 2-(4-iso­butyl­phen­yl)-2,3a-dimethyl-2,3,3a,4-tetra­hydro-1*H*-pyrrolo[1,2-*a*]benzimidazol-1-one (CSD refcode AKURII; Patil *et al.*, 2010 ▸) and 5a-*p-*tolyl-5a,5b,6,7,8,9,9a,10-octa­hydro-5*H*-isoindolo(2,1-*a*)benzimidazol-10-one – a substituted benzimidazolone ring fused with cyclo­hexane (CSD refcode ZENVUJ; Sillanpää *et al.*, 1995 ▸). From comparison of the reported structure with literature data, one can notice that the N1—C5 bond length in the title structure is shorter than in the related heterocycles CIGPEN01 and AKURII. This is related to the π-acceptor properties of the nitro­phenyl­diazenyl group.

## Synthesis and crystallization   

The synthesis of **2** was carried out according to the procedure, proposed by Gavkus *et al.*, 2012 ▸, starting from 4-nitro­aniline and **1**. The product was isolated with 87% yield and recrystallized from aceto­nitrile as ruby-red prisms. A suitable single crystal was obtained by slow cooling of the saturated solution of **2** in DMSO–aceto­nitrile mixture at 1:1 ratio.

## Refinement   

Crystal data, details of data collection and structure refinement details are summarized in Table 3 ▸. All non-H atoms, involving solvent mol­ecules, were refined anisotropically. The N—H hydrogen atom was located from a difference map and refined isotropically. The C—H hydrogen atoms were positioned geometrically and refined using a riding model.

## Supplementary Material

Crystal structure: contains datablock(s) I. DOI: 10.1107/S2056989017013937/yk2109sup1.cif

Supporting information file. DOI: 10.1107/S2056989017013937/yk2109Isup4.cdx

CCDC reference: 843227

Additional supporting information:  crystallographic information; 3D view; checkCIF report

## Figures and Tables

**Table 1:** Hydrogen-bond geometry (Å, °) *Cg*1 is the centroid of the C11–C16 ring.

*D*—H⋯*A*	*D*—H	H⋯*A*	*D*⋯*A*	*D*—H⋯*A*
N1—H1*N*⋯O1^i^	0.88 (2)	2.04 (2)	2.8550 (15)	154.9 (19)
C2—H2*A*⋯N1*SB* ^ii^	0.99	2.52	3.43 (2)	153
C2—H2*B*⋯O1*S* ^iii^	0.99	2.43	3.348 (14)	154
C2—H2*B*⋯N1*SB* ^iii^	0.99	2.43	3.37 (3)	158
C2*S*—H2*SA*⋯*Cg*1^ii^	0.96	2.93	3.766 (3)	146

Symmetry codes: (i) 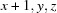; (ii) 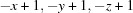; (iii) 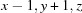.

**Table 2:** Experimental and calculated parameters of π–π inter­actions in **2**

Rings	Energy (kcal mol^−1^)	Inter­centroid distance (Å)		Inter­planar distance (Å)		Ring offset (Å)		Angle (°)	
		exp	calcd	exp	calcd	exp	calcd	exp	calcd
Benzimidazole/4-nitro­phen­yl	−16.48	3.8290 (9)	3.876	3.5025 (12)	3.485	1.547 (2)	1.698	23.814 (5)	25.977
**p*-*Tol­yl	−3.07	4.3241 (13)	4.807	3.628 (2)	3.740	2.353 (3)	3.018	32.963 (3)	38.920

**Table 3:** Experimental details

Crystal data	
Chemical formula	C_23_H_19_N_5_O_3_·0.58C_2_H_6_OS·0.42C_2_H_3_N
*M*_r_	476.17
Crystal system, space group	Triclinic, *P* 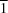
Temperature (K)	100
*a*, *b*, *c* (Å)	7.1755 (5), 10.7013 (8), 16.2586 (11)
α, β, γ (°)	86.072 (3), 78.868 (2), 73.222 (3)
*V* (Å^3^)	1172.71 (14)
*Z*	2
Radiation type	Mo *K*α
μ (mm^−1^)	0.14
Crystal size (mm)	0.27 × 0.22 × 0.21

Data collection	
Diffractometer	Bruker APEXII CCD area detector
Absorption correction	Multi-scan (*SADABS*; Bruker, 2008 ▸)
*T*_min_, *T*_max_	0.963, 0.971
No. of measured, independent and observed [*I* > 2σ(*I*)] reflections	15024, 6820, 5126
*R*_int_	0.024
(sin θ/λ)_max_ (Å^−1^)	0.704

Refinement	
*R*[*F* ^2^ > 2σ(*F* ^2^)], *wR*(*F* ^2^), *S*	0.050, 0.134, 1.00
No. of reflections	6820
No. of parameters	350
No. of restraints	25
H-atom treatment	H atoms treated by a mixture of independent and constrained refinement
Δρ_max_, Δρ_min_ (e Å^−3^)	0.46, −0.50

Computer programs: *APEX2* (Bruker, 2011 ▸), *SAINT* (Bruker, 2009 ▸), *SHELXS97* and *SHELXL97* (Sheldrick, 2008 ▸), *OLEX2* (Dolomanov *et al.* and 2009 ▸), *publCIF* (Westrip, 2010 ▸).

## supplementary crystallographic information

### Crystal data

**Table:**

C_23_H_19_N_5_O_3_·0.58C_2_H_6_OS·0.42C_2_H_3_N	*Z* = 2
*M**_r_* = 476.17	*F*(000) = 499
Triclinic, *P*1	*D*_x_ = 1.349 Mg m^−^^3^
Hall symbol: -P 1	Mo *K*α radiation, λ = 0.71073 Å
*a* = 7.1755 (5) Å	Cell parameters from 679 reflections
*b* = 10.7013 (8) Å	θ = 3–30°
*c* = 16.2586 (11) Å	µ = 0.14 mm^−^^1^
α = 86.072 (3)°	*T* = 100 K
β = 78.868 (2)°	Prism, red
γ = 73.222 (3)°	0.27 × 0.22 × 0.21 mm
*V* = 1172.71 (14) Å^3^	

### Data collection

**Table:**

Bruker APEXII CCD area detector diffractometer	6820 independent reflections
Radiation source: fine-focus sealed tube	5126 reflections with *I* > 2σ(*I*)
Graphite monochromator	*R*_int_ = 0.024
ω scans	θ_max_ = 30.0°, θ_min_ = 2.0°
Absorption correction: multi-scan (SADABS; Bruker, 2008)	*h* = −10→10
*T*_min_ = 0.963, *T*_max_ = 0.971	*k* = −15→14
15024 measured reflections	*l* = −22→22

### Refinement

**Table:**

Refinement on *F*^2^	Primary atom site location: structure-invariant direct methods
Least-squares matrix: full	Secondary atom site location: difference Fourier map
*R*[*F*^2^ > 2σ(*F*^2^)] = 0.050	Hydrogen site location: inferred from neighbouring sites
*wR*(*F*^2^) = 0.134	H atoms treated by a mixture of independent and constrained refinement
*S* = 1.00	*w* = 1/[σ^2^(*F*_o_^2^) + (0.0632*P*)^2^ + 0.525*P*] where *P* = (*F*_o_^2^ + 2*F*_c_^2^)/3
6820 reflections	(Δ/σ)_max_ < 0.001
350 parameters	Δρ_max_ = 0.46 e Å^−^^3^
25 restraints	Δρ_min_ = −0.50 e Å^−^^3^

### Special details

**Table:**

Geometry. All esds (except the esd in the dihedral angle between two l.s. planes) are estimated using the full covariance matrix. The cell esds are taken into account individually in the estimation of esds in distances, angles and torsion angles; correlations between esds in cell parameters are only used when they are defined by crystal symmetry. An approximate (isotropic) treatment of cell esds is used for estimating esds involving l.s. planes.
Refinement. Refinement of F^2^ against ALL reflections. The weighted R-factor wR and goodness of fit S are based on F^2^, conventional R-factors R are based on F, with F set to zero for negative F^2^. The threshold expression of F^2^ > 2sigma(F^2^) is used only for calculating R-factors(gt) etc. and is not relevant to the choice of reflections for refinement. R-factors based on F^2^ are statistically about twice as large as those based on F, and R- factors based on ALL data will be even larger.

### Fractional atomic coordinates and isotropic or equivalent isotropic displacement parameters (Å^2^)

**Table:**

	*x*	*y*	*z*	*U*_iso_*/*U*_eq_	Occ. (<1)
O1	0.17180 (14)	0.76220 (11)	0.30872 (7)	0.0229 (2)	
O2	0.2794 (2)	0.02811 (13)	−0.10319 (9)	0.0436 (4)	
O3	0.5660 (2)	0.01862 (13)	−0.17906 (8)	0.0428 (3)	
N1	0.76692 (17)	0.79781 (11)	0.29851 (7)	0.0152 (2)	
H1N	0.876 (3)	0.810 (2)	0.3093 (13)	0.035 (5)*	
N2	0.48636 (16)	0.73034 (11)	0.33286 (7)	0.0141 (2)	
N3	0.70223 (19)	0.43649 (12)	0.08643 (7)	0.0200 (2)	
N4	0.54027 (19)	0.40565 (12)	0.10212 (7)	0.0203 (2)	
N5	0.4347 (3)	0.05826 (14)	−0.11836 (9)	0.0330 (3)	
C1	0.28738 (19)	0.79333 (14)	0.34383 (8)	0.0168 (3)	
C2	0.2492 (2)	0.90348 (15)	0.40379 (9)	0.0205 (3)	
H2A	0.2166	0.8749	0.4627	0.025*	
H2B	0.1403	0.9792	0.3914	0.025*	
C3	0.44855 (19)	0.93659 (13)	0.38670 (9)	0.0168 (3)	
H3A	0.4570	0.9966	0.3379	0.020*	
H3B	0.4686	0.9767	0.4362	0.020*	
C4	0.59959 (18)	0.80249 (13)	0.36856 (8)	0.0135 (2)	
C5	0.77798 (19)	0.70403 (13)	0.24332 (8)	0.0145 (2)	
C6	0.9259 (2)	0.65182 (14)	0.17582 (8)	0.0181 (3)	
H6A	1.0456	0.6763	0.1641	0.022*	
C7	0.8930 (2)	0.56275 (14)	0.12611 (8)	0.0189 (3)	
H7A	0.9922	0.5261	0.0797	0.023*	
C8	0.7174 (2)	0.52528 (13)	0.14252 (8)	0.0172 (3)	
C9	0.5690 (2)	0.57522 (13)	0.21261 (8)	0.0157 (3)	
H9A	0.4504	0.5494	0.2253	0.019*	
C10	0.60410 (18)	0.66223 (13)	0.26114 (8)	0.0138 (2)	
C11	0.6773 (2)	0.73681 (13)	0.44615 (8)	0.0166 (3)	
C12	0.8005 (2)	0.78988 (14)	0.48057 (8)	0.0169 (3)	
H12A	0.8305	0.8668	0.4564	0.020*	
C13	0.8798 (2)	0.73152 (15)	0.54974 (9)	0.0215 (3)	
H13A	0.9633	0.7692	0.5724	0.026*	
C14	0.8394 (3)	0.61915 (17)	0.58628 (11)	0.0332 (4)	
C15	0.7196 (4)	0.5662 (2)	0.55122 (14)	0.0515 (6)	
H15A	0.6917	0.4885	0.5749	0.062*	
C16	0.6385 (3)	0.62415 (19)	0.48177 (12)	0.0401 (5)	
H16A	0.5560	0.5858	0.4589	0.048*	
C17	0.9234 (4)	0.5562 (2)	0.66234 (13)	0.0463 (5)	
H17A	0.9225	0.4646	0.6666	0.069*	
H17B	0.8426	0.6022	0.7129	0.069*	
H17C	1.0596	0.5610	0.6568	0.069*	
C18	0.5271 (2)	0.31506 (14)	0.04503 (9)	0.0214 (3)	
C19	0.6755 (3)	0.26357 (16)	−0.02325 (9)	0.0289 (3)	
H19A	0.7968	0.2859	−0.0327	0.035*	
C20	0.6442 (3)	0.17971 (17)	−0.07702 (10)	0.0320 (4)	
H20A	0.7424	0.1453	−0.1243	0.038*	
C21	0.4679 (3)	0.14689 (15)	−0.06083 (10)	0.0275 (3)	
C22	0.3205 (3)	0.19436 (15)	0.00692 (10)	0.0273 (3)	
H22A	0.2010	0.1696	0.0168	0.033*	
C23	0.3516 (2)	0.27924 (15)	0.06024 (10)	0.0249 (3)	
H23A	0.2527	0.3131	0.1074	0.030*	
S1S	0.77680 (10)	0.20694 (10)	0.31863 (5)	0.0258 (3)	0.585 (3)
O1S	0.801 (2)	0.0884 (12)	0.3766 (7)	0.0334 (17)	0.585 (3)
C1S	0.9536 (5)	0.1596 (4)	0.2247 (2)	0.0525 (11)	0.585 (3)
H1SA	1.0805	0.1651	0.2321	0.079*	0.585 (3)
H1SB	0.9113	0.2167	0.1795	0.079*	0.585 (3)
H1SC	0.9634	0.0715	0.2118	0.079*	0.585 (3)
C2S	0.5644 (4)	0.2154 (3)	0.2752 (2)	0.0233 (6)	0.585 (3)
H2SA	0.4507	0.2204	0.3185	0.035*	0.585 (3)
H2SB	0.5891	0.1397	0.2419	0.035*	0.585 (3)
H2SC	0.5403	0.2923	0.2402	0.035*	0.585 (3)
N1SB	0.804 (5)	0.107 (2)	0.3821 (14)	0.036 (3)	0.415 (3)
C1SB	0.7529 (9)	0.1442 (7)	0.3226 (4)	0.0437 (11)	0.415 (3)
C2SB	0.685 (2)	0.2100 (8)	0.2476 (6)	0.101 (4)	0.415 (3)
H2S1	0.7907	0.2424	0.2135	0.151*	0.415 (3)
H2S2	0.5678	0.2835	0.2639	0.151*	0.415 (3)
H2S3	0.6526	0.1483	0.2149	0.151*	0.415 (3)

### Atomic displacement parameters (Å^2^)

**Table:**

	*U*^11^	*U*^22^	*U*^33^	*U*^12^	*U*^13^	*U*^23^
O1	0.0135 (5)	0.0311 (6)	0.0268 (5)	−0.0084 (4)	−0.0049 (4)	−0.0074 (4)
O2	0.0700 (10)	0.0360 (7)	0.0395 (7)	−0.0281 (7)	−0.0255 (7)	0.0013 (6)
O3	0.0742 (10)	0.0321 (7)	0.0254 (6)	−0.0148 (7)	−0.0157 (6)	−0.0063 (5)
N1	0.0126 (5)	0.0217 (6)	0.0142 (5)	−0.0085 (4)	−0.0030 (4)	−0.0009 (4)
N2	0.0124 (5)	0.0180 (5)	0.0143 (5)	−0.0068 (4)	−0.0037 (4)	−0.0023 (4)
N3	0.0273 (6)	0.0175 (6)	0.0159 (5)	−0.0057 (5)	−0.0064 (5)	0.0002 (4)
N4	0.0296 (6)	0.0182 (6)	0.0152 (5)	−0.0078 (5)	−0.0074 (5)	−0.0006 (4)
N5	0.0608 (10)	0.0216 (7)	0.0228 (7)	−0.0140 (7)	−0.0196 (7)	0.0026 (5)
C1	0.0131 (6)	0.0214 (7)	0.0169 (6)	−0.0066 (5)	−0.0021 (5)	−0.0014 (5)
C2	0.0149 (6)	0.0245 (7)	0.0225 (7)	−0.0052 (5)	−0.0022 (5)	−0.0077 (6)
C3	0.0175 (6)	0.0181 (6)	0.0171 (6)	−0.0062 (5)	−0.0059 (5)	−0.0025 (5)
C4	0.0124 (5)	0.0178 (6)	0.0135 (6)	−0.0080 (5)	−0.0038 (4)	−0.0009 (5)
C5	0.0137 (6)	0.0177 (6)	0.0133 (6)	−0.0048 (5)	−0.0052 (4)	0.0019 (5)
C6	0.0145 (6)	0.0250 (7)	0.0156 (6)	−0.0073 (5)	−0.0031 (5)	0.0016 (5)
C7	0.0182 (6)	0.0228 (7)	0.0133 (6)	−0.0029 (5)	−0.0015 (5)	−0.0007 (5)
C8	0.0211 (6)	0.0170 (6)	0.0140 (6)	−0.0047 (5)	−0.0054 (5)	0.0003 (5)
C9	0.0168 (6)	0.0171 (6)	0.0146 (6)	−0.0060 (5)	−0.0046 (5)	0.0005 (5)
C10	0.0127 (5)	0.0163 (6)	0.0127 (6)	−0.0041 (5)	−0.0031 (4)	0.0007 (5)
C11	0.0176 (6)	0.0192 (6)	0.0153 (6)	−0.0069 (5)	−0.0060 (5)	0.0004 (5)
C12	0.0178 (6)	0.0194 (6)	0.0162 (6)	−0.0082 (5)	−0.0049 (5)	−0.0001 (5)
C13	0.0246 (7)	0.0247 (7)	0.0197 (7)	−0.0098 (6)	−0.0106 (5)	0.0001 (5)
C14	0.0520 (11)	0.0288 (8)	0.0300 (8)	−0.0189 (8)	−0.0262 (8)	0.0108 (7)
C15	0.0889 (17)	0.0467 (11)	0.0498 (12)	−0.0506 (12)	−0.0491 (12)	0.0314 (10)
C16	0.0626 (12)	0.0416 (10)	0.0396 (10)	−0.0394 (10)	−0.0355 (9)	0.0208 (8)
C17	0.0751 (15)	0.0387 (10)	0.0420 (11)	−0.0259 (10)	−0.0427 (11)	0.0187 (8)
C18	0.0346 (8)	0.0171 (6)	0.0144 (6)	−0.0080 (6)	−0.0088 (6)	0.0016 (5)
C19	0.0455 (10)	0.0283 (8)	0.0166 (7)	−0.0192 (7)	−0.0002 (6)	−0.0026 (6)
C20	0.0537 (11)	0.0285 (8)	0.0158 (7)	−0.0175 (8)	−0.0014 (7)	−0.0033 (6)
C21	0.0514 (10)	0.0172 (7)	0.0191 (7)	−0.0114 (7)	−0.0175 (7)	0.0026 (5)
C22	0.0363 (9)	0.0213 (7)	0.0295 (8)	−0.0092 (6)	−0.0172 (7)	0.0017 (6)
C23	0.0309 (8)	0.0211 (7)	0.0239 (7)	−0.0053 (6)	−0.0104 (6)	−0.0023 (6)
S1S	0.0239 (4)	0.0302 (5)	0.0298 (4)	−0.0135 (3)	−0.0133 (3)	0.0054 (3)
O1S	0.0293 (17)	0.039 (4)	0.038 (3)	−0.015 (2)	−0.021 (2)	0.020 (3)
C1S	0.0248 (15)	0.059 (2)	0.056 (2)	−0.0019 (15)	0.0106 (14)	0.0246 (19)
C2S	0.0258 (13)	0.0254 (14)	0.0225 (14)	−0.0121 (11)	−0.0089 (10)	0.0073 (10)
N1SB	0.040 (4)	0.040 (5)	0.035 (3)	−0.018 (3)	−0.017 (3)	0.001 (3)
C1SB	0.062 (3)	0.036 (3)	0.049 (3)	−0.027 (2)	−0.027 (2)	0.009 (2)
C2SB	0.212 (13)	0.060 (5)	0.073 (6)	−0.066 (7)	−0.095 (7)	0.028 (4)

### Geometric parameters (Å, º)

**Table:**

O1—C1	1.2219 (16)	C13—H13A	0.9500
O2—N5	1.224 (2)	C14—C15	1.378 (2)
O3—N5	1.228 (2)	C14—C17	1.512 (2)
N1—C5	1.3670 (17)	C15—C16	1.396 (2)
N1—C4	1.4787 (17)	C15—H15A	0.9500
N1—H1N	0.88 (2)	C16—H16A	0.9500
N2—C1	1.3737 (17)	C17—H17A	0.9800
N2—C10	1.4091 (17)	C17—H17B	0.9800
N2—C4	1.4828 (16)	C17—H17C	0.9800
N3—N4	1.2728 (18)	C18—C23	1.393 (2)
N3—C8	1.4012 (18)	C18—C19	1.399 (2)
N4—C18	1.4214 (18)	C19—C20	1.386 (2)
N5—C21	1.4727 (19)	C19—H19A	0.9500
C1—C2	1.5109 (19)	C20—C21	1.381 (3)
C2—C3	1.5409 (19)	C20—H20A	0.9500
C2—H2A	0.9900	C21—C22	1.381 (2)
C2—H2B	0.9900	C22—C23	1.388 (2)
C3—C4	1.5354 (19)	C22—H22A	0.9500
C3—H3A	0.9900	C23—H23A	0.9500
C3—H3B	0.9900	S1S—O1S	1.517 (11)
C4—C11	1.5231 (18)	S1S—C2S	1.776 (3)
C5—C6	1.3912 (19)	S1S—C1S	1.785 (4)
C5—C10	1.4162 (18)	C1S—H1SA	0.9599
C6—C7	1.388 (2)	C1S—H1SB	0.9601
C6—H6A	0.9500	C1S—H1SC	0.9600
C7—C8	1.4021 (19)	C2S—H2SA	0.9598
C7—H7A	0.9500	C2S—H2SB	0.9600
C8—C9	1.4160 (19)	C2S—H2SC	0.9601
C9—C10	1.3659 (18)	N1SB—C1SB	1.108 (16)
C9—H9A	0.9500	C1SB—C2SB	1.460 (8)
C11—C16	1.379 (2)	C2SB—H2SB	1.1778
C11—C12	1.3936 (18)	C2SB—H2SC	1.1738
C12—C13	1.3874 (19)	C2SB—H2S1	0.9800
C12—H12A	0.9500	C2SB—H2S2	0.9800
C13—C14	1.386 (2)	C2SB—H2S3	0.9800
			
C5—N1—C4	109.49 (10)	C15—C14—C13	117.95 (14)
C5—N1—H1N	120.1 (14)	C15—C14—C17	120.77 (16)
C4—N1—H1N	118.6 (13)	C13—C14—C17	121.28 (15)
C1—N2—C10	126.55 (11)	C14—C15—C16	121.51 (16)
C1—N2—C4	112.88 (11)	C14—C15—H15A	119.2
C10—N2—C4	110.00 (10)	C16—C15—H15A	119.2
N4—N3—C8	114.52 (12)	C11—C16—C15	120.37 (15)
N3—N4—C18	113.95 (12)	C11—C16—H16A	119.8
O2—N5—O3	123.77 (14)	C15—C16—H16A	119.8
O2—N5—C21	118.60 (16)	C14—C17—H17A	109.5
O3—N5—C21	117.63 (16)	C14—C17—H17B	109.5
O1—C1—N2	123.73 (13)	H17A—C17—H17B	109.5
O1—C1—C2	129.41 (13)	C14—C17—H17C	109.5
N2—C1—C2	106.85 (11)	H17A—C17—H17C	109.5
C1—C2—C3	102.37 (11)	H17B—C17—H17C	109.5
C1—C2—H2A	111.3	C23—C18—C19	120.23 (14)
C3—C2—H2A	111.3	C23—C18—N4	115.52 (14)
C1—C2—H2B	111.3	C19—C18—N4	124.25 (14)
C3—C2—H2B	111.3	C20—C19—C18	119.51 (16)
H2A—C2—H2B	109.2	C20—C19—H19A	120.2
C4—C3—C2	102.76 (11)	C18—C19—H19A	120.2
C4—C3—H3A	111.2	C21—C20—C19	118.97 (16)
C2—C3—H3A	111.2	C21—C20—H20A	120.5
C4—C3—H3B	111.2	C19—C20—H20A	120.5
C2—C3—H3B	111.2	C22—C21—C20	122.72 (14)
H3A—C3—H3B	109.1	C22—C21—N5	118.46 (15)
N1—C4—N2	101.19 (10)	C20—C21—N5	118.82 (16)
N1—C4—C11	109.81 (10)	C21—C22—C23	118.16 (15)
N2—C4—C11	113.51 (11)	C21—C22—H22A	120.9
N1—C4—C3	116.20 (11)	C23—C22—H22A	120.9
N2—C4—C3	102.46 (10)	C22—C23—C18	120.39 (15)
C11—C4—C3	112.96 (11)	C22—C23—H23A	119.8
N1—C5—C6	129.84 (12)	C18—C23—H23A	119.8
N1—C5—C10	110.23 (11)	O1S—S1S—C2S	105.3 (5)
C6—C5—C10	119.90 (12)	O1S—S1S—C1S	106.9 (6)
C7—C6—C5	117.82 (12)	C2S—S1S—C1S	96.38 (17)
C7—C6—H6A	121.1	S1S—C1S—H1SA	109.8
C5—C6—H6A	121.1	S1S—C1S—H1SB	109.3
C6—C7—C8	121.87 (13)	H1SA—C1S—H1SB	109.5
C6—C7—H7A	119.1	S1S—C1S—H1SC	109.3
C8—C7—H7A	119.1	H1SA—C1S—H1SC	109.5
N3—C8—C7	115.87 (12)	H1SB—C1S—H1SC	109.5
N3—C8—C9	123.65 (12)	S1S—C2S—H2SA	111.1
C7—C8—C9	120.48 (12)	S1S—C2S—H2SB	109.6
C10—C9—C8	116.92 (12)	H2SA—C2S—H2SB	109.5
C10—C9—H9A	121.5	S1S—C2S—H2SC	107.8
C8—C9—H9A	121.5	H2SA—C2S—H2SC	109.5
C9—C10—N2	130.64 (12)	H2SB—C2S—H2SC	109.5
C9—C10—C5	122.94 (12)	N1SB—C1SB—C2SB	172.7 (15)
N2—C10—C5	106.39 (11)	C1SB—C2SB—H2SB	93.7
C16—C11—C12	118.43 (13)	C1SB—C2SB—H2SC	130.4
C16—C11—C4	123.11 (12)	H2SB—C2SB—H2SC	83.6
C12—C11—C4	118.40 (12)	C1SB—C2SB—H2S1	109.5
C13—C12—C11	120.64 (13)	C1SB—C2SB—H2S2	109.5
C13—C12—H12A	119.7	H2S1—C2SB—H2S2	109.5
C11—C12—H12A	119.7	C1SB—C2SB—H2S3	109.5
C14—C13—C12	121.10 (13)	H2S1—C2SB—H2S3	109.5
C14—C13—H13A	119.5	H2S2—C2SB—H2S3	109.5
C12—C13—H13A	119.5		
			
C8—N3—N4—C18	−179.71 (11)	N1—C5—C10—C9	175.62 (12)
C10—N2—C1—O1	27.6 (2)	C6—C5—C10—C9	−2.8 (2)
C4—N2—C1—O1	168.43 (13)	N1—C5—C10—N2	−2.58 (14)
C10—N2—C1—C2	−151.40 (13)	C6—C5—C10—N2	179.04 (11)
C4—N2—C1—C2	−10.58 (15)	N1—C4—C11—C16	−115.12 (17)
O1—C1—C2—C3	−150.17 (15)	N2—C4—C11—C16	−2.7 (2)
N2—C1—C2—C3	28.76 (14)	C3—C4—C11—C16	113.40 (17)
C1—C2—C3—C4	−35.31 (13)	N1—C4—C11—C12	61.98 (16)
C5—N1—C4—N2	−16.28 (13)	N2—C4—C11—C12	174.42 (12)
C5—N1—C4—C11	103.95 (12)	C3—C4—C11—C12	−69.49 (15)
C5—N1—C4—C3	−126.30 (12)	C16—C11—C12—C13	−0.9 (2)
C1—N2—C4—N1	−132.55 (11)	C4—C11—C12—C13	−178.10 (13)
C10—N2—C4—N1	14.76 (13)	C11—C12—C13—C14	0.2 (2)
C1—N2—C4—C11	109.88 (13)	C12—C13—C14—C15	0.7 (3)
C10—N2—C4—C11	−102.81 (12)	C12—C13—C14—C17	−179.21 (18)
C1—N2—C4—C3	−12.25 (14)	C13—C14—C15—C16	−0.9 (4)
C10—N2—C4—C3	135.06 (11)	C17—C14—C15—C16	179.0 (2)
C2—C3—C4—N1	138.38 (11)	C12—C11—C16—C15	0.7 (3)
C2—C3—C4—N2	29.10 (12)	C4—C11—C16—C15	177.80 (19)
C2—C3—C4—C11	−93.40 (12)	C14—C15—C16—C11	0.2 (4)
C4—N1—C5—C6	−169.39 (13)	N3—N4—C18—C23	−179.91 (12)
C4—N1—C5—C10	12.44 (14)	N3—N4—C18—C19	−0.5 (2)
N1—C5—C6—C7	−175.73 (13)	C23—C18—C19—C20	1.9 (2)
C10—C5—C6—C7	2.29 (19)	N4—C18—C19—C20	−177.45 (14)
C5—C6—C7—C8	−0.1 (2)	C18—C19—C20—C21	−1.3 (3)
N4—N3—C8—C7	−179.04 (12)	C19—C20—C21—C22	0.1 (3)
N4—N3—C8—C9	1.62 (19)	C19—C20—C21—N5	179.70 (15)
C6—C7—C8—N3	178.86 (12)	O2—N5—C21—C22	−1.3 (2)
C6—C7—C8—C9	−1.8 (2)	O3—N5—C21—C22	178.84 (14)
N3—C8—C9—C10	−179.31 (12)	O2—N5—C21—C20	179.07 (15)
C7—C8—C9—C10	1.4 (2)	O3—N5—C21—C20	−0.8 (2)
C8—C9—C10—N2	178.58 (13)	C20—C21—C22—C23	0.5 (2)
C8—C9—C10—C5	0.86 (19)	N5—C21—C22—C23	−179.14 (13)
C1—N2—C10—C9	−44.5 (2)	C21—C22—C23—C18	0.2 (2)
C4—N2—C10—C9	173.73 (13)	C19—C18—C23—C22	−1.3 (2)
C1—N2—C10—C5	133.46 (13)	N4—C18—C23—C22	178.05 (13)
C4—N2—C10—C5	−8.26 (14)		

### Hydrogen-bond geometry (Å, º)

Cg1 is the centroid of the C11–C16 ring.

**Table:**

*D*—H···*A*	*D*—H	H···*A*	*D*···*A*	*D*—H···*A*
N1—H1*N*···O1^i^	0.88 (2)	2.04 (2)	2.8550 (15)	154.9 (19)
C2—H2*A*···N1*SB*^ii^	0.99	2.52	3.43 (2)	153
C2—H2*B*···O1*S*^iii^	0.99	2.43	3.348 (14)	154
C2—H2*B*···N1*SB*^iii^	0.99	2.43	3.37 (3)	158
C2*S*—H2*SA*···*Cg*1^ii^	0.96	2.93	3.766 (3)	146

Symmetry codes: (i) *x*+1, *y*, *z*; (ii) −*x*+1, −*y*+1, −*z*+1; (iii) *x*−1, *y*+1, *z*.
